# A Comparison of Women’s and Men’s Web-Based Information-Seeking Behaviors About Gender-Related Health Information: Web-Based Survey Study of a Stratified German Sample

**DOI:** 10.2196/43897

**Published:** 2023-05-17

**Authors:** Elena Link, Eva Baumann

**Affiliations:** 1 Department of Communication Johannes Gutenberg University Mainz Mainz Germany; 2 Department of Journalism and Communcation Research Hanover University of Music, Drama and Media Hanover Germany

**Keywords:** health information-seeking behavior, HISB, gender, sex, planned risk information seeking model, subjective norms, risk perceptions, affective risk responses, attitudes toward seeking, perceived seeking control

## Abstract

**Background:**

Gender-sensitive approaches to health communication aim to integrate gender perspectives at all levels of communication, as an individual’s biological sex and socially assigned gender identity have an impact on whether and how one acquires what type of health information. Due to the fast and low-cost opportunity to search for a wide range of information, the internet seems to be a particularly suitable place for gender-related health information about diseases of sex-specific organs and diseases where biological differences are associated with different health risks.

**Objective:**

This study aims to inform gender-related information provision and acquisition in 2 ways. The first objective was to provide a theory-driven analysis of web-based health information–seeking behavior (HISB) regarding gender-related issues. Therefore, the Planned Risk Information Seeking Model (PRISM), which is one of the most integrative models of HISB, was adapted and applied. Second, we asked for gender-specific motivational determinants of gender-related web-based HISB comparing the predictors in the groups of women and men.

**Methods:**

Data from a stratified web-based survey of the German population (N=3000) explained gender-related web-based HISB and influencing patterns comparing women and men. The applicability of PRISM to gender-related web-based HISB was tested using structural equation modeling and a multigroup comparison.

**Results:**

The results revealed PRISM as an effective framework for explaining gender-related web-based HISB. The model accounted for 28.8% of the variance in gender-related web-based HISB. Gender-related subjective norms provided the most crucial explanatory power, followed by perceived seeking control. The multigroup comparison revealed differences in the model’s explanatory power and the relevance of predictors of gender-related web-based HISB. The share of explained variances of web-based HISB is higher in men than in women. For men, norms were a more relevant promoting factor, whereas web-based HISB of women was more strongly associated with perceived seeking control.

**Conclusions:**

The results are crucial for gender-sensitive targeting strategies and suggest gender-related health information interventions that address gender-related subjective norms. Furthermore, programs (eg, web-based learning units) should be developed and offered to improve individuals’ (perceived) abilities to perform web-based searches for health information, as those with higher control beliefs are more likely to access web-based information.

## Introduction

### Relevance of Examining Gender-Related Health Information–Seeking Behavior

Individuals’ web-based health information–seeking behaviors (HISBs) are perceived as capable of enhancing one’s knowledge and empowerment, as the internet provides the opportunity for a fast and low-cost search for a wide range of health information [1-7]. Vice versa, the varying ways in which individuals search for health information can be linked to varying health outcomes and disparities in health achievements [8]. Whether and how one acquires health information can be associated with different needs, concerns, and abilities that depend, among others, on personal characteristics such as gender [9-11]. It is not only a person’s sexual category as the biological facet of gender that influences an individual’s web-based HISB. Furthermore, women and men act differently according to facets of gender, that is, their socially assigned gender identities and internalized gender roles, which are sociocultural constructs tied to, but not similar to their biological sex [12,13]. Focusing on HISB-related facets of gender, it is empirically supported that women are more concerned with health and are more frequent health information seekers than men [1,14].

To address the health gaps resulting from beliefs and behaviors influenced by the different biological and social gender facets [10,15], gender-sensitive approaches to health communication aim to integrate gender perspectives at all levels of communication [8,16-19]. Offering an extensive range of information and a low-threshold opportunity for targeted searches, the internet appears to be a particularly suitable place for gender-related health information. Information on diseases of sex-specific organs is just as needed and accessible as gender-related information about a disease where biological differences cause different risk levels [15].

To ensure appropriate gender-related information provision and acquisition, research is needed to describe and explain women’s and men’s search for gender-related health information and its gender-specific predictors. Against this background, the overall aim of this study was to contribute to a deeper understanding of gender-related HISB. Therefore, we designed a study with 2 purposes. First, we go beyond existing studies focusing on the frequency of women’s and men’s web-based HISB [1,20] and aim for a theory-driven analysis of web-based HISB regarding gender-related issues. Therefore, one of the latest and most general HISB models—the Planned Risk Information Seeking Model (PRISM) [21]—was adapted and applied for web-based HISB regarding gender-related information. Second, we asked for gender-specific motivational determinants of gender-related web-based HISB comparing the predictors in women and men. This knowledge will allow us to evaluate the need for gender-related targeting strategies that can reduce health inequalities between women and men [22].

### Theoretical Background

#### Theory-Driven Modeling of Gender-Related Web-Based HISB

HISB is a purposive acquisition of information about one’s health, health promotion activities, health risks, and diseases from selected information carriers such as the internet [10,23]. To explain individuals’ web-based HISB, several well-established models of HISB, such as the PRISM [21], the Risk Information Seeking and Processing Model (RISP) [24,25], the Theory of Motivated Information Management (TMIM) [26-28], and the Comprehensive Model of Information Seeking (CMIS) [23], have already been adapted for web-based searches for health information [7,29-34]. Compared with these models, PRISM is one of the latest models that integrates models such as the RISP, the TMIM, the CMIS, and the Theory of Planned Behavior (TPB) [35,36]. It was developed to explain information-seeking intentions focusing on general cognitive and sociopsychological factors that motivate HISB (the study by Kahlor [21] provides a more detailed description of the PRISM).

The PRISM posits the importance of 7 individual-level predictors that motivate HISB intention. As postulated in the TPB, attitudes toward a behavior such as HISB (hypothesis [H] 1), seeking-related subjective norms (H2), and the perception of one’s behavioral control over seeking (H3) [35,36] are considered in the PRISM to explain information-seeking intentions. Attitudes toward information seeking refer to one’s instrumental and experiential evaluation of HISB [21]. Subjective norms subsume injunctive and descriptive norms describing motivations to seek information because of striving for conformity, a perceived social pressure, or the fear of social punishment [21,37]. Perceived seeking control refers to one’s perceived ability to perform HISB [7,21,35].

In line with RISP [24], the intention to search for health information is assumed to be associated with an individual’s health-related risk perceptions and affective risk responses. Risk perceptions refer to individuals’ perceived susceptibility and severity, indicating whether a health risk is relevant to the individual [21,24]. This judgmental-oriented dimension is not a direct predictor of HISB but is considered an antecedent to emotional responses to risks (H4) [38]. These affective evaluations can lead to more active HISB, serving as a strategy to cope with negative affective responses (H5).

In the RISP tradition, perceived knowledge and knowledge insufficiency are the key motives for seeking information [24,38]. Perceived knowledge describes the actual state of knowledge, whereas knowledge insufficiency captures the desired level of knowledge. The relevance of both is rooted in the sufficiency threshold by Chaiken [39], which describes the level of knowledge to adequately deal with a health problem or risk [21]. In this context, it is important to consider the expression of the desired level of information to deal with health problems and risks. In line with RISP and PRISM, it can be proposed that the desired level of information (“information insufficiency”) is a driver of HISB (H6). Perceived knowledge insufficiency is itself determined by negative affective risk responses (H7), attitudes toward information seeking (H8), perceived seeking control (H9), and subjective norms (H10). Perceived knowledge is affected by attitudes toward seeking (H11), perceived seeking control (H12), and subjective norms (H13).

Among the predictors of HISB, all are theorized to be positively related to HISB intentions across contexts, sources, and various issues [21,40]. In line with this assumption, the current state of research confirms that PRISM can be applied to explain web-based information searches [7,31] and is valid across a variety of health and environmental issues [7]. However, the paths for web-based HISB are understudied compared with HISB in general, as outlined in a meta-analysis by Wang et al [7].

This study aimed to test the assumptions of PRISM for gender-related health information but focused on the frequency of actual web-based HISB instead of the intention to web-based HISB. The frequency captures interest-oriented habitual tendencies to acquire information about specific issues, such as gender-related health information, and provides a more conservative test of PRISM as the intention is a strong predictor of, but not equal to, performed HISB. Postulates of the PRISM are assumed to apply to the frequency of HISB as other models of HISB, such as the RISP and CMIS integrated into PRISM, capture not only HISB intentions but also tendencies to seek information or actual HISB (eg, [7,24,29]). In particular, the RISP, understood as the origin of PRISM, is based on a broader understanding of HISB.

**Figure 1:**
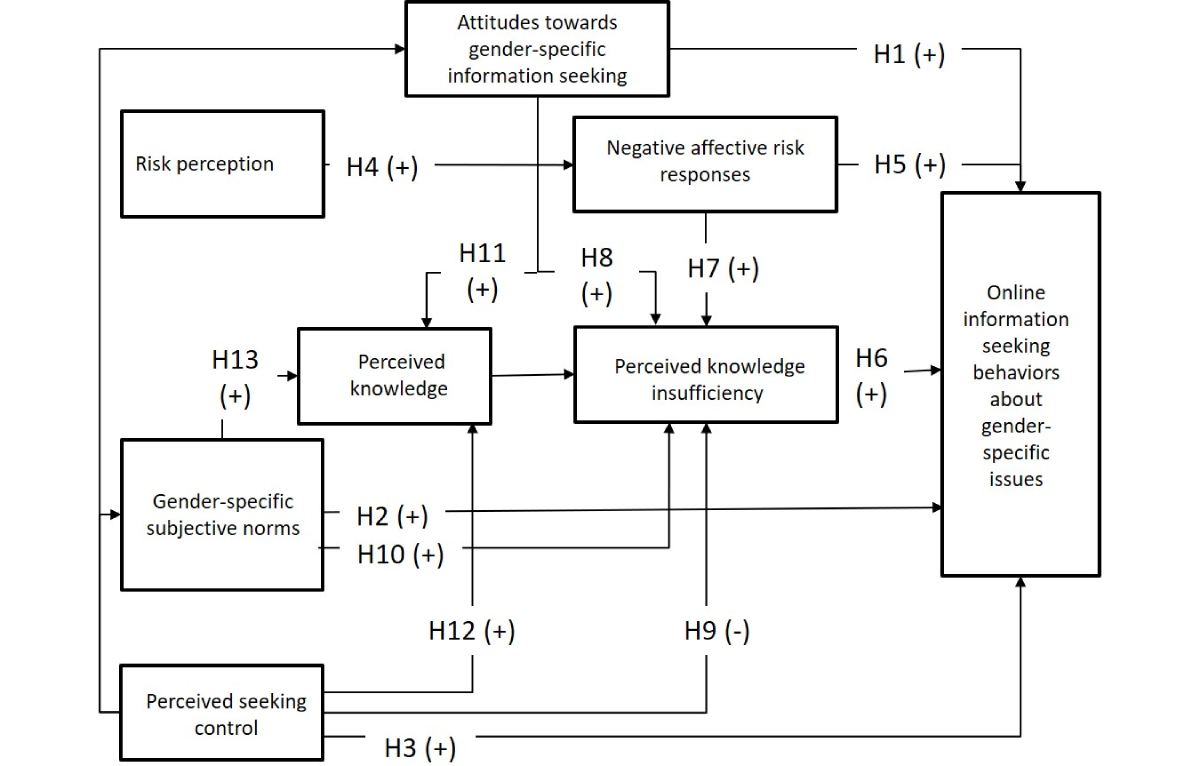
Derived hypotheses to predict web-based health information-seeking behavior about gender-specific issues. H: hypothesis.

In line with the original PRISM, the proposed model paths (H1 to H13) to predict gender-related web-based HISB are illustrated in Figure 1.

#### Theoretical Foundation of Gender Differences

PRISM neglects the relevance of individual characteristics such as gender because there is no fully developed theoretical rationale for their inclusion [21,40]. To further evaluate and question this postulate regarding gender, we aimed to provide an overview of the suggested theoretical foundations for why meta-analyses conclude that men are less likely to actively seek health information on the web than similarly situated women [1,7,16,20,22,41-43].

Suggested reasons for gender differences in web-based HISB include gender socialization resulting in socially bound identities, roles, or self-concepts [13,20,44-46]. In the process of socialization, individuals learn about shared beliefs, cultural values, and practices regarding the negative and positive qualities of men and women and incorporate these masculine and feminine attributes into their gender identity [14,44,47]. Masculine attributes focus on achievement, control, and power, whereas feminine attributes focus on being more socially engaged. Thus, feminine attributes include empathy, nurturance, focusing on others, social relationships, and to be more anxious and uncertain [44]. In line with stereotypes for masculinity and femininity, being concerned with health or seeking medical advice are seen as feminine qualities and something men do not (need to) engage in [1,14,41,42,48]. Further research suggests that gender determines the perceived ability to perform web-based HISB [41,48].

To summarize, these theoretical assumptions call into question whether it is still tenable for PRISM to neglect the relevance of gender and suggest integrating gender into PRISM.

#### Gender Influences on Gender-Related Web-Based HISB

To integrate gender into PRISM, we refer to the existing findings and assumptions about how gender impacts intentional and attitude-related processes relevant to (web-based) HISB [23,24,41,49]. However, the research is limited and often focuses on the biological dimension of gender (male vs female individuals).

Regarding the concept of attitudes toward information seeking considered in PRISM, we refer to the assumptions of CMIS [23,29]. It posits that gender is related to the perceived information carrier utility, understood as the perceived usefulness of information, similar to the attitudes considered in PRISM. Although the findings of Basynat et al [29] could not support the idea that gender determines the perceived utility of the internet, Bidmon and Terlutter [41] showed that women have more positive attitudes toward the internet than men.

Regarding risk perception as one of the central predictors of HISB according to PRISM, men are known to perceive a lower level of risk and feel less worried about risks than women [20,24]. On the basis of gender socialization, the decline in the risk perceptions of men is attributed to identity protection. This motive leads individuals to perceive risks in a way that improves their standing with the groups to which they belong [20,50]. Risk perception and HISB are assumed to pose a threat to one’s masculinity, as it requires an individual’s acknowledgment of uncertainty and vulnerability [20].

Gender identities are also closely tied to norms of how women or men behave and their perceptions of how women or men ought to behave [13,14]. In this regard, it is assumed that masculine norms can discourage male individuals from seeking information, whereas female individuals hold more pronounced norms supporting their HISB [13,51,52]. Extant research that asked for gender differences in normative influence on web-based HISB is rare but found that normative influences were only weakly pronounced across women and men [41]. The literature focusing on norms within the gender groups to which one belongs is lacking.

The already introduced attributes of masculine and feminine gender identity can also influence individuals perceived seeking control to perform web-based HISB. In line with the achieving and controlling attributes of men and higher levels of uncertainty attributed to women, Bidmon and Terlutter [41] found that women displayed a lower perceived seeking control than men.

To conclude, recent research suggests that gender may result in different influence patterns of gender-specific web-based HISB. However, the state of research examining how gender (socialization) may affect attitudes toward information seeking, seeking-related subjective norms, one’s perceived state of knowledge or knowledge insufficiency, risk perceptions, and perceived seeking control is relatively scarce. This overview underscores that it is highly relevant to focus on gender-related behaviors and investigate the underlying mechanisms and gender-specific predictors of web-based HISB. To substantiate this assumption, we propose a comparison of gender-specific influence patterns. Therefore, we developed the following research question: *How does gender impact PRISM’s assumptions with regard to explaining gender-related web-based HISB?*

## Methods

### Recruitment Procedure and Participants

We conducted a web-based survey (N=3000) with a sample stratified by sex assigned at birth, age (18-74 years), education, and region for the German population. The participants were recruited via the German web-based access panel Respondi and Bilendi. Data were collected in 2022. The mean age of the respondents was 46.13 (SD 15.36) years. Half of the respondents (1502/3000, 50.07%) were female individuals, 33.67% (1010/3000) had completed junior high school at most, 32.33% (970/3000) had a general certificate of upper secondary education, and 34% (1020/3000) had at least a university entrance qualification. Furthermore, 16.03% (481/3000) of the respondents reported having a migration background (Table 1).

All questions were mandatory; therefore, missing data were not an issue. Furthermore, 3 questions to identify invalid responses were included (ie, attention checks by selecting specific responses in an item battery), which led to an early end and exclusion of respondents by the panel provider. Prescreening the data set regarding streamlining behavior or reply patterns revealed no random responses.

**Table 1:** Overview of the subsamples.

			Subsamples						Difference (test statistics)	
			Female (n=1502)		Male (n=1498)		Total (N=3000)			
Age (years), mean (SD)			41.95 (14.62)		50.32 (14.95)		46.13 (15.36)		*F*_2998_=240.20; *P*<.001; η^2^=0.074	
**Education, n (%)**										χ^2^_2_=21.5; Cramer V=.085; *P*<.001
	Low	446 (29.7)		564 (37.7)		1010 (33.7)				
	Medium	520 (34.6)		450 (30)		970 (32.3)				
	High	536 (35.7)		484 (32.3)		1020 (34)				
Migration background, n (%)			261 (17.4)		220 (14.7)		481 (16)		χ^2^_1_=4.0; Cramer V=.042; *P*=.07	
Gender-related web-based health information–seeking behaviors, mean (SD)			2.12 (1.02)		1.86 (0.95)		1.99 (1.00)		*F*_2998_=50.30; *P*<.001; η^2^=0.017	
Attitudes toward information seeking, mean (SD)			3.41 (0.86)		3.32 (0.87)		3.37 (0.86)		*F*_2998_=7.89; *P*=.005; η^2^=0.003	
Subjective gender-related norms, mean (SD)			2.95 (0.84)		2.62 (0.84)		2.79 (0.85)		*F*_2998_=120.44; *P*<.001; η^2^=0.039	
Perceived seeking control, mean (SD)			2.93 (1.03)		3.04 (1.00)		2.98 (1.01)		*F*_2998_=9.18; *P*=.002; η^2^=0.003	
Perceived knowledge, mean (SD)			50.87 (24.37)		47.37 (25.38)		49.12 (24.9)		*F*_2998_=14.90; *P*<.001; η^2^=0.005	
Perceived knowledge insufficiency, mean (SD)			69.30 (19.91)		66.29 (21.39)		67.80 (20.71)		*F*_2998_=15.87; *P*<.001; η^2^=0.005	
Risk perception, mean (SD)			4.60 (2.11)		4.69 (2.11)		4.65 (2.12)		*F*_2998_=1.44; *P*=.23; η^2^=0.000	
Negative affective risk response, mean (SD)			2.61 (0.93)		2.41 (0.94)		2.51 (0.94)		*F*_2998_=34.01; *P*<.001; η^2^=0.011	

### Ethical Considerations, Informed Consent, and Participation

This type of data collection, according to the German standards, was considered exempt from ethics approval. However, steps were taken to warrant the principles of ethical research and to protect the personal rights of study participants. All participants were informed about the strictly scientific purpose of the research, the expected duration, and procedures. They were asked to provide informed consent at the beginning of the survey and were informed about their right to cancel their participation and the confidentiality of their responses. No personally identifiable information was collected. The commercial market research company that recruited and compensated the participants was certified in accordance with the International Organization for Standardization 20252 and followed the International Chamber of Commerce and European Society for Opinion and Marketing Research International Code on Market, Opinion and Social Research.

### Measures

#### Overview

All items for the questionnaire were adapted from prior literature. The established measures were translated into German using a team translation approach, comparing, adjusting, and pretesting 2 independent translations of a graduate translator and a research team member. The item wording and the zero-order correlations are presented in Tables S1 and S2 in Multimedia Appendix 1 [21,31,38,53]. Table 2 presents an overview of the data fit of the measurement models. Each measurement model was evaluated for their reliability and fit to the data using the following fit indices: root mean square error of approximation (RMSEA), comparative fit index (CFI), and standardized root mean square residual (SRMR).

**Table 2:** Overview of the measurement models fit to the data.

Measurement models	Fit to the data					
	Cronbach α (95% CI)	Chi-square (*df*)	*P* value	CFI^a^	RMSEA^b^ (95% CI)	SRMR^c^
Attitudes toward information seeking	.94 (0.93-0.94)	41.5 (14)	<.001	0.996	0.026 (0.020-0.032)	0.011
Perceived seeking control	.90 (0.89-0.90)	7.6 (2)	.02	0.998	0.030 (0.015-0.047)	0.008
Negative affective risk response	.89 (0.89-0.90)	0.2 (1)	.66	1.00	0.00 (0.00-0.03)	0.003
Social norms	.83 (0.82-0.84)	53.3 (4)	<.001	0.984	0.064 (0.053-0.076)	0.022

^a^CFI: comparative fit index.

^b^RMSEA: root mean square error of approximation.

^c^SRMR: standardized root mean square residual.

#### Gender-Related Web-Based Health Information Seeking

To describe the respondents’ frequency of web-based searches about gender-related issues, a single item was used (mean 1.99, SD 1.00; Table S1 in Multimedia Appendix 1 includes the wording of the question). Before the query, respondents were given a definition of gender-related health information, which defined this information as “addressing your gender” and gave examples such as breast and prostate cancer, specific screening services, or forms of contraception. The participants reported their responses on a 5-point Likert-type scale ranging from never (1) to very often (5).

#### Attitudes Toward Information Seeking

To assess individuals’ evaluation to acquire gender-related health information, we used a 7-item measure applying PRISM that has been used in previous studies (eg, [21]). The 5-point semantic differential items asked the participants whether information seeking was bad or good, harmful or beneficial, or unhelp or helpful. The measurement model showed a good fit to the data (Cronbach α=.94; mean 3.37, SD 0.86; Table 2; Table S1 in Multimedia Appendix 1).

#### Subjective Gender-Related Norms

To measure subjective gender-related norms, we adapted a measure from the study by Venkatesh et al [53]. The measure consisted of 5 items describing injunctive and descriptive norms. The reference group of norms was automatically adjusted to the gender of the respondents (“Most women/most men/most nonbinary persons I know...”). The 5 items rated on a 5-point Likert-type scale (1=strongly disagree to 5=strongly agree) were combined into a measurement model (Cronbach α=.83; mean 2.79, SD 0.85; Table 2).

#### Perceived Seeking Control

In line with past research [21,54,55], the respondents’ perceived ability to seek gender-related information on the internet was measured using a 4-item measure that considered whether the respondents know how and where to find and evaluate gender-related health information on the web. The participants reported their responses on a 5-point Likert-type scale ranging from 1 (strongly disagree) to 5 (strongly agree). The data of the measurement model were evaluated as satisfactory (Cronbach α=.90; mean 2.98, SD 1.01; Table 2).

#### Perceived Knowledge and Perceived Knowledge Insufficiency

Consistent with the study by Kahlor et al [21], perceived knowledge and perceived knowledge insufficiency were measured by asking the respondents to describe their actual level of knowledge (mean 49.12, SD 24.94) and their desired level of knowledge about gender-related health issues (mean 67.80, SD 20.71) on scales ranging from 0 to 100.

#### Risk Perception

Following the study by Link et al [31], we measured risk perception by asking for the susceptibility and the severity of an illness [21,56]. Both items were measured on a Likert-type scale ranging from 1 (not at all) to 10 (extremely; Cronbach α=.74; mean 4.65, SD 2.12).

#### Negative Affective Risk Responses

An adapted measurement by Yang and Kahlor [38] was used to assess the respondents’ negative affective risk responses. In both cases, the participants were asked to indicate whether they experienced emotions such as worry on a 5-point semantic differential scale. The data of the measurement model were evaluated as satisfactory (Cronbach α=.89; mean 2.51, SD 0.94; Table 2).

#### Gender

The respondents were asked to indicate whether they were male, female, or nonbinary. In addition, there was the possibility of making open statements to further elaborate on the information. All the respondents were female (1502/3000, 50.07%) or male (1498/3000, 49.93%) individuals. Furthermore, we asked about gender roles using a 5-item measure that can be used to assess self-ascribed masculinity or femininity [57]. The unidimensional structure was supported (Cronbach α=.97; mean 2.96, SD 1.34). The (biological) gender query and the scale assessing gender roles were highly correlated (*r*=0.838; *P*≤.001). Thus, we used the dichotomous gender query as the basis for the multigroup comparison.

### Data Analysis

To test our hypotheses and answer our research question, we used latent variable structural equation modeling in R (R Foundation for Statistical Computing). We used 2-step modeling. In the first step, all measurement models were verified, and their data fit and measurement invariance were evaluated. The data fit of each measurement model was evaluated as satisfactory (Table 2), and the measurement invariance appeared justifiable to satisfying for multigroup comparison (Table S3 in Multimedia Appendix 1). In the second step, the structural invariance was determined by comparing the unconstrained and constrained models using χ^2^ and fit statistics, and the structural model was tested.

## Results

### Predictors of Gender-Related Web-Based HISB

The adapted PRISM (H1-H13) to explain gender-related web-based HISB showed a satisfactory model fit (χ^2^_233_=823.6; *P*≤.001; CFI=0.981; RMSEA=0.029, 95% CI 0.027-0.031; SRMR=0.029). As the other indices had very satisfactory levels, the significant χ^2^ test was attributed to the sample size (Hoelter critical N [CN]=814.60). In total, the model accounted for 28.8% of the variance in gender-related web-based HISB. Results of the hypotheses tests for the single paths (standardized β coefficients and their significance) are reported in Table 3. Overall, 12 out of the 13 hypotheses were confirmed. The single path that could not be confirmed was the association between knowledge insufficiency and gender-related web-based HISB (H6).

**Table 3:** Overview of the hypotheses and outcomes.

Proposed path	Standardized β coefficients		
	Total sample	Women	Men
H^a^1: attitude toward seeking → gender-related web-based HISB^b^ (+)	.101^c^	.100^c^	.108^c^
H2: gender-related seeking-related subjective norms → gender-related web-based HISB (+)	.255^c^	.200^c^	.330^c^
H3: perceived seeking control → gender-related web-based HISB (+)	.206^c^	.288^c^	.235^c^
H4: risk perceptions → negative affective risk responses (−)	.354^c^	.369^c^	.351^c^
H5: negative affective risk response → gender-related web-based HISB (+)	.109^c^	.089^c^	.108^c^
H6: perceived knowledge insufficiency → gender-related web-based HISB (+)	-.007	.034	.035
H7: negative affective risk response → perceived knowledge insufficiency (+)	.045^d^	.061^d^	.034
H8: attitude toward seeking → perceived knowledge insufficiency (+)	.059^d^	.037	.080^d^
H9: perceived seeking control → perceived knowledge insufficiency (−)	-.091^c^	-.117^c^	-.073
H10: gender-related subjective norms → perceived knowledge insufficiency (+)	.116^c^	.146^c^	.075^d^
H11: attitude toward seeking → perceived knowledge (+)	.045^d^	.035	.050
H12: perceived seeking control → perceived knowledge (+)	.378^c^	.408^c^	.354^c^
H13: gender-related subjective norms → perceived knowledge (+)	.192^c^	.150^c^	.237^c^

^a^H: hypothesis.

^b^HISB: health information–seeking behavior.

^c^*P*<.001.

^d^*P*<.05.

### Group Differences Between the Predictors of Gender-Related Web-Based HISB

The research question asked for differences in the predictors of web-based HISB between women and men. A comparisons of the unconstrained and constrained models showed that both models fit the data fairly well (unconstrained: χ^2^_470_=1128.9; *P*≤.001; CFI=0.979; RMSEA=0.031, 95% CI 0.029-0.033; SRMR=0.035; CN=1143.90 and constrained: χ^2^_519_=1584.8; *P*≤.001; CFI=0.964; RMSEA=0.037, 95% CI 0.035-0.039; SRMR=0.040), but the χ^2^ difference test indicated that the models were not equivalent (Δχ^2^_49_=514.4; *P*≤.001). The unconstrained model was superior, implying that the path coefficients varied among men and women.

A comparison of the power of the model for women and men showed that the model accounted for a higher level of variance for men’s gender-related web-based HISB compared with women’s web-based HISB (women: *R*^2^=0.233 vs men: *R*^2^=0.307; Δ*R*^2^=0.075).

Looking at single paths (Table 3), we first introduce stronger associations for men than for women. We found more robust relationships between gender-related subjective norms and web-based HISB (H2; men: β=.330; *P*≤.001 and women: β=.200; *P*≤.001), negative affective risk responses and web-based HISB (H5; men: β=.108; *P*≤.001 and women: β=.089; *P*≤.001), subjective norms and perceived knowledge (H13; men: β=.237; *P*≤.001 and women: β=.150; *P*≤.001) for men than women. Although in the male group, attitudes toward seeking had a weak but significant association with perceived knowledge insufficiency, this path was not significant in the female group (H8; men: β=.080; *P*=.02 and women: β=.037; *P*=.25).

Stronger associations for women compared with men were found for the paths between gender-related subjective norms and perceived knowledge insufficiency (H10: men: β=.075; *P*=.02 and women: β=.146; *P*≤.001), perceived seeking control and gender-related web-based HISB (H3: men: β=.235; *P*≤.001 and women: β=.288; *P*≤.001), risk perceptions and negative affective risk responses (H4: men: β=.351; *P*≤.001 and women: β=.369; *P*≤.001), and perceived seeking control and perceived knowledge (H12: men: β=.354; *P*≤.001 and women: β=.408; *P*≤.001). Other differences were found in the relationships between negative affective risk responses and perceived knowledge insufficiency (H7; men: β=.034; *P*=.21 and women: β=.061; *P*=.03) and between perceived seeking control and perceived knowledge insufficiency (H9; men: β=−.073, *P*=.05 and women: β=−.12; *P*≤.001). In both cases, only the paths for the female individuals were significant.

Consistent across both groups is the strength of the association between attitudes toward seeking and gender-related web-based HISB (H1; men: β=.108; *P*≤.001 and women: β=.100; *P*≤.001), the missing significant paths between perceived knowledge insufficiency and gender-related web-based HISB (H6; men: β=.035; *P*=.13 and women: β=.034; *P*=.14), and the missing path between attitudes toward seeking and perceived knowledge (H11; men: β=.050; *P*=.11 and women: β=.035; *P*=.25).

## Discussion

### Key Findings About the Predictors of Gender-Related Web-Based HISB

Against gender-sensitive approaches stressing the relevance of gender-related information provision and acquisition, we applied PRISM [21] to provide a theoretically sound analysis of predictors of web-based HISB of gender-related issues. In line with previous research showing that PRISM is valid across contexts, sources, and a variety of issues [7,21,31,40], our results reveal that PRISM is also an effective framework for predicting web-based HISB regarding gender-related health information. However, it should be noted that gender-related web-based HISB was rare in the examined sample of the German population (mean 1.99, SD 1.00).

Focusing on the predictors of web-based HISB, our study adds to an understudied association between subjective norms and web-based HISB [7]. Gender-related seeking-related subjective norms had the highest explanatory power for web-based HISB, which is in line with international studies that stress the role of normative influences for HISB in general [25]. More pronounced seeking-related norms among women and men were associated with a more frequent web-based seeking for gender-related information. The findings stress that subjective norms are a crucial constituent of the PRISM to explain web-based HISB and suggest that the effect of subjective norms holds equally across different topics [7]. Compared with studies showing a rather weak impact of injunctive and descriptive norms (eg, [31]), this study and its findings might be shaped by the fact that we addressed gender groups as reference group instead of significant others such as family and friends. This is a unique feature of the study that we estimated as relevant to consider social determinants of gender-related HISB.

The second most important predictor was perceived seeking control, followed by negative affective risk responses and attitudes toward seeking. All predictors were associated with a more frequent gender-related web-based HISB. The relatively high importance of perceived seeking control may be attributed to the exceptionally crucial role of being able to find reliable and accurate health information in the digital age, where a vast array of information of varying quality is available, and it can be challenging to evaluate [31,58]. Related problems were often the subject of public discourse, most recently during the COVID-19 pandemic under the term “infodemic” [59,60]. It is assumed that people were more likely to be confronted with misleading, inconsistent, and partly unreliable information during this time. Furthermore, being confronted with potentially misleading and overwhelming information may have influenced their perception of their capabilities and the importance of these abilities for implementing a web-based search. This finding is consistent with a comparatively low expression of individuals’ perceived seeking control (Table 2).

Compared with subjective norms and perceived seeking control, risk perceptions, affective risk responses, and attitudes toward information seeking were observed to be only weakly related to gender-related web-based HISB. The low correlation between attitudes and web-based search should be interpreted against a heterogeneous state of research. The meta-analysis by Wang et al [7] described the number of studies examining the role of attitudes as insufficient. There are studies showing a rather strong association (eg, [21,31]) and studies showing nonsignificant results [55]. Regarding the low impact of affective risk responses, this finding might be a result of the channel examined [61] and may be attributed to individuals’ relatively low-risk perceptions and affective risk responses [31,62]. Extant research suggests that risk perceptions and affective risk responses are less relevant on the web than on other channels [61]. In addition, our focus on the frequency of web-based HISB instead of web-based HISB intention might influence a lower relevance of severity, susceptibility, and related affective responses.

The missing significant association between information insufficiency and gender-related HISB is in line with studies that found no relation between both [21,25,63]. Such findings raise 2 critical questions: first, in which contexts, related to which sources or issues does information insufficiency contribute to a better understanding of (web-based) HISB and second, whether excluding information insufficiency from the PRISM benefits the development of a more parsimonious model of HISB.

### Key Findings About the Impact of Gender on Gender-Related Web-Based HISB

As gender identities are assumed to influence how individuals acquire information [10,15], we also included masculinity and femininity gender roles in our measures. As these were highly correlated to biological gender categories, we examined differences between predictors determining web-based HISB for women and men. The multigroup comparison revealed differences in the explanatory power of the PRISM predictors of web-based HISB. In general, PRISM was able to explain more variance within the sample of men compared with women. Referring to the single predictors, most differences refer to the effect size of the associations but not their significance. We found 2 major patterns of association distinguishing women and men.

First, our findings suggest that women’s gender-related web-based HISB is more strongly affected by their perceived seeking control. This pattern refers not only to the direct association between perceived seeking control and gender-related HISB. Furthermore, more pronounced capabilities to seek health information on the web are related to a higher perceived level of knowledge and a lower perceived level of information insufficiency. Thus, perceived seeking control is an enabling factor for women, in particular, to search for gender-related information and become more knowledgeable. This result adds to the existing findings that women have lower levels of perceived seeking control [41] and stresses that it is crucial to overcome this barrier of empowerment through health communication interventions.

Second, for men, subjective seeking-related norms were identified as more influential for their gender-related web-based HISB than for women. Thus, if men believe that other men search for health information and that it is expected of men to seek health information, they more frequently search for gender-related information. Their subjective norms were also more strongly associated with their knowledge level but less related to their level of information insufficiency. Once again, the main contrast to other findings showing only a little impact of norms [41] is the gender group as a reference group for normative beliefs. Thus, masculine norms can not only discourage male individuals from seeking information [51] but also motivate them to turn to the internet to search for gender-related health information.

As a third key finding, we want to highlight that the postulated differences in risk perceptions [20,24,50] did not manifest in major differences between the web-based HISB of women and men. Independent of the different perceptions of risks and potential measures of identity protection, the risk perceptions of women and men were a determinant of negative risk responses, which were associated with a slightly more frequent web-based HISB for gender-related information. Negative affective responses were found to have a slightly stronger influence on gender-related web-based HISB among men compared with women.

Overall, the results of the multigroup comparison suggest that the relative importance of predictors of gender-related web-based HISB differs depending on individuals’ gender. Therefore, considering gender more thoroughly is a valuable extension for theory-based modeling of web-based HISB and provides insights for designing gender-sensitive interventions.

### Limitations and Resulting Tasks for Future Research

Although this study informs about gender-related web-based HISB using a comparative approach for women and men, the following limitations of the study need to be considered. First, the measure used for gender-related web-based HISB can be assessed as insufficient. We asked for habitual behavior instead of an intention to use gender-related information in the future, which is an adaption of PRISM. This should be assessed critically with regard to causal statements, as outlined in more detail below. Furthermore, we used only a self-constructed single item, which might be problematic because the term gender-related information is rather complicated, and it cannot be ignored that the term evokes different associations. Future research should test whether the findings hold for intentions to use gender-related HISB by applying validated measurements and distinguishing between different facets of gender-related health information. A second limitation is that our interpretations of the impact of gender are based on socialization and different facets of gender identity, without integrating an existing measure of feminine and masculine aspects of one’s gender identity into PRISM. To provide deeper insights, the different facets of gender identity should be considered more comprehensively in relation to biological gender categories and integrated within models of HISB. Third, our study was based on cross-sectional data that do not allow for causal statements. To ensure a deeper understanding of information searches, longitudinal or tracking studies are required in future research to distinguish between the predictors and outcomes of HISB. Fourth, the subsamples of women and men differed in their characteristics. In particular, we found major differences regarding their age. Therefore, we performed an additional analysis step to include age as a covariate in the multigroup comparison. The explained variance and the results of the hypotheses testing remained stable, indicating that our findings of the comparison between women and men are robust and reliable.

### Conclusions and Practical Implications

Understanding the gender-related patterns of HISB offers practical insights to policy makers, health organizations, and health care professionals and can guide them in improving health and reducing gender-related health inequalities. Strategies to provide relevant gender-related health information and disseminate it effectively in the web-based domain can benefit from these findings. First, the study showed that gender-related information is only seldomly searched for on the internet. This indicates the potential to increase interest, raise awareness for gender-related informational needs, and improve information provision in this domain. Second, the varying influential importance of predictors among women and men allows us to characterize both target audiences with greater precision and inform gender-related, or at least gender-sensitive, targeting strategies [22]. To maximize the reach of gender-related health information, interventions and campaigns should address social norms in the future, particularly when targeting male individuals. Furthermore, programs (eg, web-based learning units) should be developed and offered to improve individuals’ (perceived) abilities to perform web-based searches for health information, as those with higher control beliefs are more likely to access web-based information. Furthermore, for those who do not believe in their ability to find and acquire information, services such as seals of quality or fact checkers might provide crucial guidance, and additional communication strategies beyond web-based information are recommended.

## Appendix

Multimedia Appendix 1 Overview of the measures and measurement invariance of the measurement models.

## Abbreviations

CFI: comparative fit index
CMIS: Comprehensive Model of Information Seeking
CN: Hoelter critical N
H: hypothesis
HISB: health information–seeking behavior
PRISM: Planned Risk Information Seeking Model
RISP: Risk Information Seeking and Processing Model
RMSEA: root mean square error of approximation
SRMR: standardized root mean square residual
TMIM: Theory of Motivated Information Management
TPB: Theory of Planned Behavior

## Data Availability

The data sets generated and analyzed during this study are available from the corresponding author upon reasonable request.
