# Supplementary material for: A Comparison of Women’s and Men’s Web-Based Information-Seeking Behaviors About Gender-Related Health Information: Web-Based Survey Study of a Stratified German Sample
Source: J Med Internet Res. 2023 May 17;25:e43897. doi: 10.2196/43897 (PMC10233438; doi:10.2196/43897)
Supplement: Multimedia Appendix 1 [file jmir_v25i1e43897_app1.docx]

## Online Appendix

Table A1. Overview of the Measures

| Construct | Item wording | Source |
| --- | --- | --- |
| Gender-related information seeking | How often do you purposefully seek health information related to gender-related issues on the Internet? |  |
| Attitudes towards seeking^1^ | How do you feel about seeking health information about the gender-related issue online? Seeking health information about the gender-related issues on the Internet  AT1 - … bad or good.  AT2 - … harmful or beneficial.  AT3 - … unhelpful or helpful.  AT4 - … unproductive or productive.  AT5 - … foolish or wise.  AT6 - … note useful or useful.  AT7 - … worthless or valuable. | [21] |

| Perceived seeking control^2^ | PSC1: I know where to look for gender-related health information on the Internet.  PSC2: I know how to search for information about gender-related health issues on the Internet.  PSC3: I can readily access all the information about gender-related health issues that I need.  PSC4: When it comes to finding information about gender-related health issues, I know where to go. | [21] |
| --- | --- | --- |
| Risk perception | RP1: How likely are you to become ill in the next year?^3^  RP2: If you were to become ill in the next year, how serious do you think it would be?^4^ | [31] |
| Negative affective risk response^5^ | When you think about how you feel about your health and gender-specifics to your health:  How …  AFF1 - … worried do you feel?  AFF2 - … scared do you feel?  AFF3 - … uncertain do you feel? | [38] |
| Social norms^2^ | SN1: Most [auto-fill according to gender query: women/men/non-binary people] I know seek extensive information about their health.  SN2: Most [women/men/non-binary people] I know value gender-related health information.  SN3: [Women/Men/Non-binary people] whose opinions I value seek themselves information about their health.  SN4: Most [women/men/non-binary people] think that I should seek for information about my health.  SN5: Most [women/men/non-binary people] expects me to be informed about my health. | [53] |
| Perceived knowledge | Please rate your current knowledge about gender-related health issues on a scale of 0 to 100. Zero means knowing nothing. 100 means knowing everything you could know about the gender-related health issues. | [21] |
| Perceived knowledge insufficiency | Think of that same 0 to 100 scale again. This time, estimate how much information you need to deal adequately with gender-related health issues. How much information would be sufficient for you, that is, good enough for your purposes? | [21] |
| ^1^ measured on five-point scale ranging from 1 (negative adjective) to 5 (positive adjective)  ^2^ measured on five-point Likert-type scales ranging from 1(does not apply at all) to 5 (does apply at all)  ^3^ measured on a five-point Likert-type scale ranging from 1 (extremely unlikely) to 5 (extremely likely)  ^4^ measured on a five-point Likert-type scale ranging 1 (completely harmless) to 5 (extremely dangerous)  ^5^ measured on a five-point Likert-type scale ranging from 1 (not at all) to 5 (extremely) | | |

Table A2. Zero-order Correlations

|  | 1. | 2. | 3. | 4. | 5. | 6. | 7. |
| --- | --- | --- | --- | --- | --- | --- | --- |
| 1. Gender-specific online HISB | - |  |  |  |  |  |  |
| 2. Attitudes towards information seeking | .325*** | - |  |  |  |  |  |
| 3. Subjective gender-specific norms | .386*** | .334*** | - |  |  |  |  |
| 4. Perceived seeking control | .387*** | .426*** | .315*** | - |  |  |  |
| 5. Perceived knowledge | .337*** | .285*** | .314*** | .459*** | - |  |  |
| 6. Perceived knowledge insufficiency | .169*** | .175*** | .229*** | .161*** | .415*** | - |  |
| 7. Risk perception | .047* | .039* | .034 | -.039* | -.025 | .024 | - |
| 8. Negative affective risk responses | .124*** | .039* | .144*** | -.052* | -.071** | .037* | .285*** |
| Note. * *p* < .05, ** *p* < .01, *** *p* ≤ .001 | | | | | | | |

Table A3.

*Overview of the Measurement Invariance of the Measurement Models*

| Model | Fit to the Data | | | | Model comparison | | | |
| --- | --- | --- | --- | --- | --- | --- | --- | --- |
|  | *Χ^2^* | Df | CFI | RMSEA | Δ *Χ^2^* | Δ df | Δ p | Δ CFI |
| Attitudes towards information seeking | | | | | | | | |
| Configural Invariance | 119.80 | 28 | .994 | .047 | - | - | - | - |
| Metric  Invariance | 135.35 | 34 | .994 | .045 | 15.55 | 6 | .02 | .002 |
| Scalar  Invariance | 179.24 | 40 | .991 | .048 | 43.88 | 6 | .00 | .003 |
| Residual Invariance | 210.70 | 47 | .990 | .048 | 31.47 | 7 | .00 | .000 |
| Perceived seeking control | | | | | | | | |
| Configural Invariance | 18.37 | 4 | .998 | .049 | - | - | - | - |
| Metric  Invariance | 19.01 | 7 | .998 | .034 | .64 | 3 | .89 | .015 |
| Scalar  Invariance | 22.38 | 10 | .998 | .029 | 3.37 | 3 | .34 | .005 |
| Residual Invariance | 23.76 | 14 | .999 | .022 | 1.37 | 4 | .85 | .007 |
| Negative affective risk responses | | | | | | | | |
| Configural Invariance | 1.61 | 2 | 1.00 | .000 | - | - | - | - |
| Metric  Invariance | 3.44 | 3 | 1.00 | .010 | 1.83 | 1 | .18 | .000 |
| Scalar  Invariance | 6.29 | 5 | 1.00 | .013 | 2.85 | 2 | .24 | .000 |
| Residual Invariance | 10.78 | 8 | .999 | .015 | 4.50 | 3 | .21 | .001 |
| Social norms | | | | | | | | |
| Configural Invariance | 76.82 | 8 | .987 | .076 | - | - | - | - |
| Metric  Invariance | 100.82 | 12 | .982 | .071 | 24.84 | 4 | .000 | .005 |
| Scalar  Invariance | 516.87 | 16 | .902 | .144 | 415.05 | 4 | .000 | .080 |
| Residual Invariance | 520.07 | 21 | .902 | .126 | 3.20 | 5 | .669 | .000 |
